# Case Report: Treatment of the rare B-cell lymphoblastic lymphoma with scalp lesion using rotation flap

**DOI:** 10.3389/fonc.2023.1252512

**Published:** 2023-10-20

**Authors:** Timothy Kim, Geena Jung, Emery Buckner-Wolfson, Ryan Fatemi, Genesis Liriano, Adit Tal, Yanhua Wang, Oren Tepper, Andrew Kobets

**Affiliations:** ^1^ Departments of Neurosurgery, Montefiore Medical Center and the Albert Einstein College of Medicine, Bronx, NY, United States; ^2^ Department of Pediatric Hematology/Oncology, Montefiore Medical Center, Bronx, NY, United States; ^3^ Department of Pathology, Montefiore Medical Center, Bronx, NY, United States; ^4^ Department of Surgery (Plastic and Reconstructive Surgery), Montefiore Medical Center, Bronx, NY, United States

**Keywords:** scalp lesion, B-cell lymphoblastic lymphoma, acute lymphoblastic leukemia, rotation flap, non-Hodgkin’s lymphoma

## Abstract

**Introduction:**

Leukemia is the most frequently occurring cancer in children, and lymphoblastic lymphoma (LBL) is a rare subtype. LBL are lymphoid neoplasms of B or T cell origin and are primarily treated with chemotherapy. Although cure rates among children are excellent, these patients must be monitored for relapse. Cutaneous lesions involving B-cell LBL (B-LBL) are extremely rare and here we present a patient with a worsening B-LBL scalp mass who required radical surgical excision.

**Case report:**

A 6-year-old female patient with a history of a nontender scalp mass discovered at approximately 2-3 years of age was evaluated for resection of the nodule due to its size and treatment history. The patient was originally diagnosed with follicular lymphoma by punch biopsy; excision was successfully performed on this 4 cm lesion and upon examination of the skin biopsy did we get a diagnosis of B-LBL. Reconstruction of the scalp was done through the rotation flap method. The patient’s scalp healed well, and adjuvant chemotherapy was continued. There has been no reoccurrence.

**Discussion:**

Here we report the rarity of B-LBL cases involving extranodal involvement in the scalp. The most common reconstruction of scalp lesions has been using free flap from the anterolateral thigh (ALT) and latissimus dorsi (LD). Our case used the rotation flap, which has its functional and cosmetic benefits. The importance of monitoring this patient is emphasized due to the dangerous consequences of B-LBL relapse. Ultimately, our successful treatment and care of this rare case can be used as guidance for similar patients in the future.

## Introduction

The most frequently occurring cancer in children is leukemia, which comprises 30-40% of childhood malignancies. The symptoms can be mild or progress to further complications including death (1, 2). Therefore, symptoms should be identified early on, and treatment should ensue immediately.

One subtype of leukemia is acute lymphoblastic leukemia (ALL)/lymphoblastic lymphoma (LBL). ALL and LBL are commonly classified together due to overlapping characteristics, as they are lymphoid neoplasms of B or T cell origin occurring mostly in children (3). Treatment involves an intense and prolonged course of chemotherapy and ongoing efforts are being made to improve the cure rates while minimizing the negative side effects of therapy (4).

Although cure rates are excellent in this population, these patients must be monitored for long-term toxicities and relapse. This monitoring is especially critical, since only half of children with first relapse of ALL survive long term and this number decreases even more with later relapses (5).

LBL is already a relatively uncommon neoplasm of precursor lymphocytes, comprising only 5% of non-Hodgkin’s Lymphoma (6). Cutaneous lesions are even less frequent, appearing in less than 20% of LBL cases (7). B-LBL with only skin involvement is extremely rare and there are only 38 other cases documented in the literature (8). Here, we present the case of a LBL manifesting as a worsening scalp mass which required radical surgical excision.

## Case report

**Figure 1:**
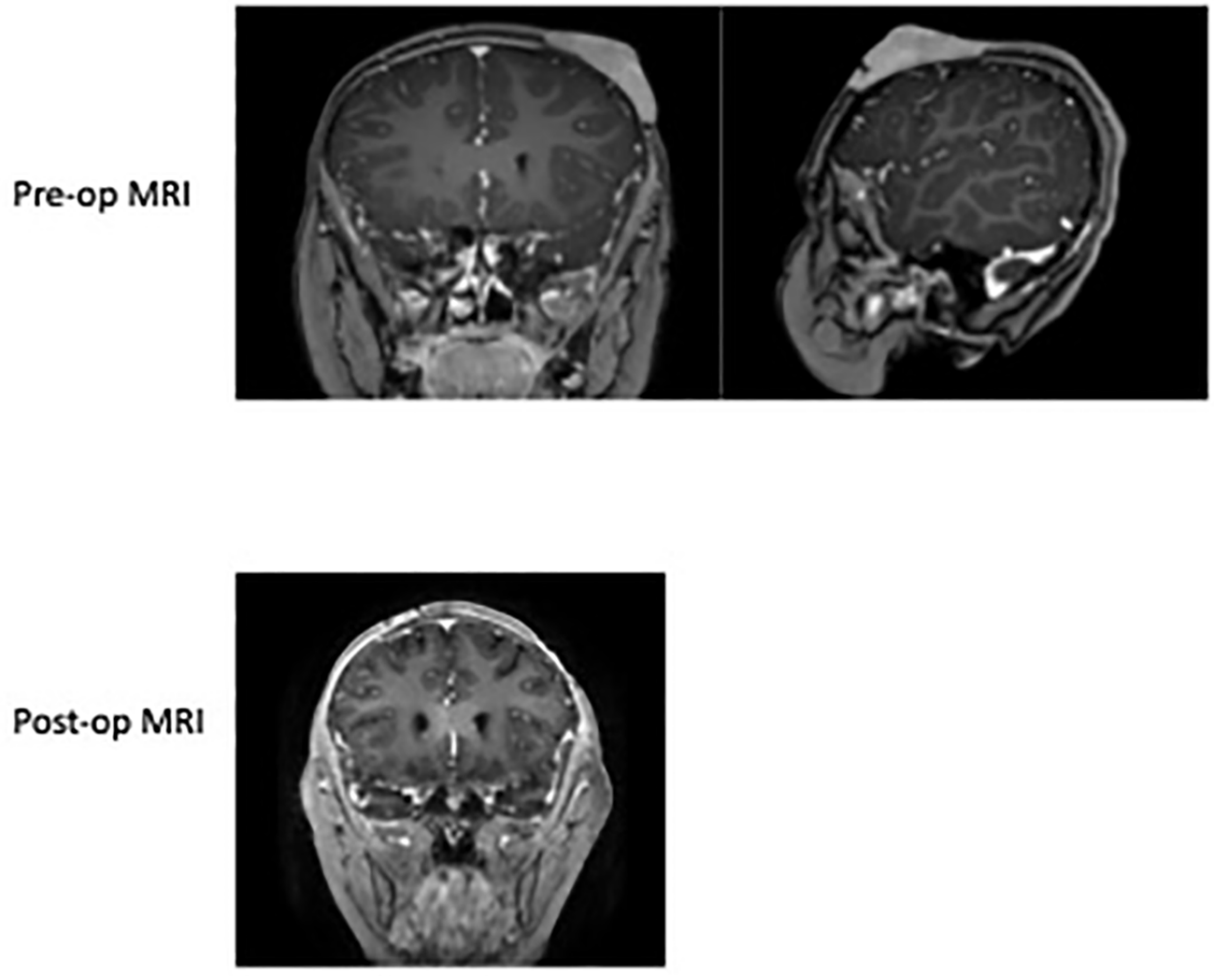
Pre-op MRI of the skull 2022 demonstrating the left anterior scalp lesion of 4.0 cm x 2.7 cm x 2.9 cm soft tissue scalp mass overlying the left frontal lobe size prior to resection. Post-op MRI of brain in 2022 showing normal curvature of the scalp after excision and a left parietal scalp postsurgical defect with mild associated enhancement, which is overall nonspecific. No abnormal parenchymal or meningeal enhancement.

A 6-year-old female presented with a history of a nontender scalp mass that was first noticed as a single nodule at approximately 2-3 years of age. She was initially being monitored by her pediatrician, and was noted to have waxing and waning erythema and enlargement. She was thus referred to the pediatric hematology/oncology clinic for further workup. An ultrasound (US) of the scalp obtained in May 2021 showed a hypervascular lesion measuring 2.7 x 0.5 cm x 2.1 cm that was consistent with a hemangioma. Multidisciplinary referral to dermatology was done for clinical monitoring and the case was discussed in vascular tumor board. Upon frequent follow up and repeat US the lesion continued to enlarge, which was unusual for a hemangioma. Thus, a punch biopsy was obtained which demonstrated a mature B cell lymphoma of follicular germinal center origin; atypical cells were positive for CD10, CD20, BCL2, and negative for CD3, CD4, CD5, CD8, CD30, CD56, MUM1, TIA1, BCL-1, and BCL6. She had no other systemic symptoms and denied fever, weight loss, night sweats, or other lymphadenopathy. Staging was subsequently done which included MRI of the disease site, PET/CT, and CXR to evaluate for mediastinal mass. MRI showed 4.0 cm x 2.7 cm x 2.9 cm soft issue scalp mass overlying the left frontal lobe, inseparable from the outer table and replacing the subcutaneous fat. The lesion enhanced, showed restricted diffusion, and had areas of susceptibility representing calcification and punctate hemorrhage possibly secondary to a recent biopsy (**Figure 1**). PET/CT showed increased uptake in the scalp mass, and tonsillar and thymic uptake, but no other avidity suggesting additional disease. She was classified as stage 1; surgery was discussed as a curative option, so she was referred to neurosurgery for surgical excision.

**Figure 2:**
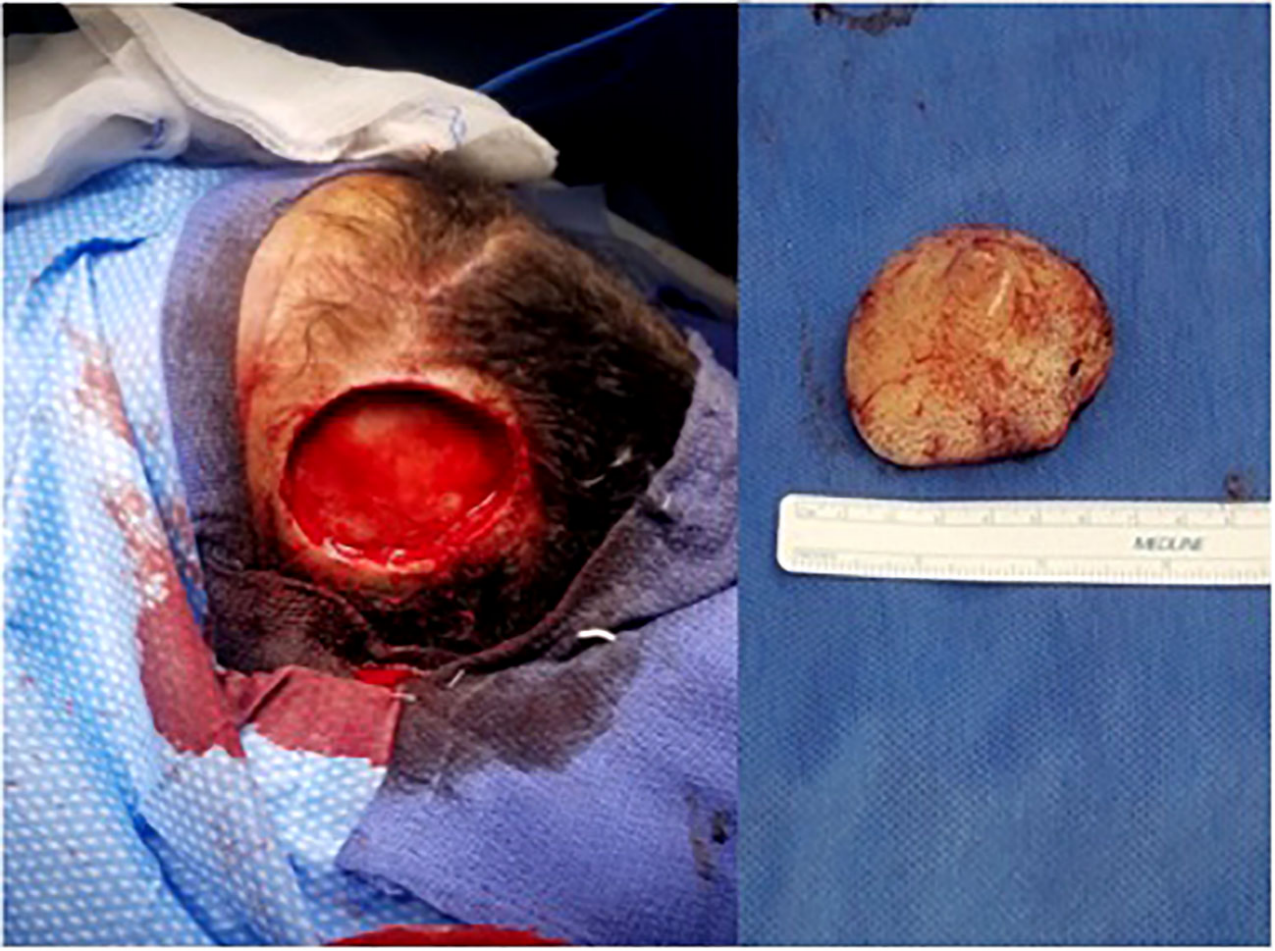
Image in the operating room of wound after the 4 x 4 cm lesion (right) was excised.

At the time of our evaluation, the lesion was about 4 cm, eroding the scalp with a firm consistency and minimal mobility over the skull. Given the large size of the lesion and the expected defect which would result from complete removal, we planned to coordinate care with our plastic surgery team. The excision of the left frontal scalp lesion was performed under general anesthesia. The skin was opened circumferentially around the tumor, and it elevated without issue with clean bone underneath. All skin edges were hemostased without issue and no palpable tumor remained. This excision left an approximate 4 cm diameter circular defect in the left anterior scalp with no bony defect (**Figure 2**). Once the mass was excised, the plastic surgery team performed a large fasciocutaneous flap from posterior scalp with supply from the left occipital artery. A large flap rotated off the posterior vessels was mobilized with another flap from the right frontoparietal region adjacent to the defect, allowing transfer of the tissues over the defect in the subgaleal plane. The flap was elevated and well vascularized with these rotational components both posteriorly and contralaterally. The entire defect was closed with excellent attention to cosmesis. The patient was stable postoperatively and had an uncomplicated course before being discharged home in 2 days.

**Figure 3:**
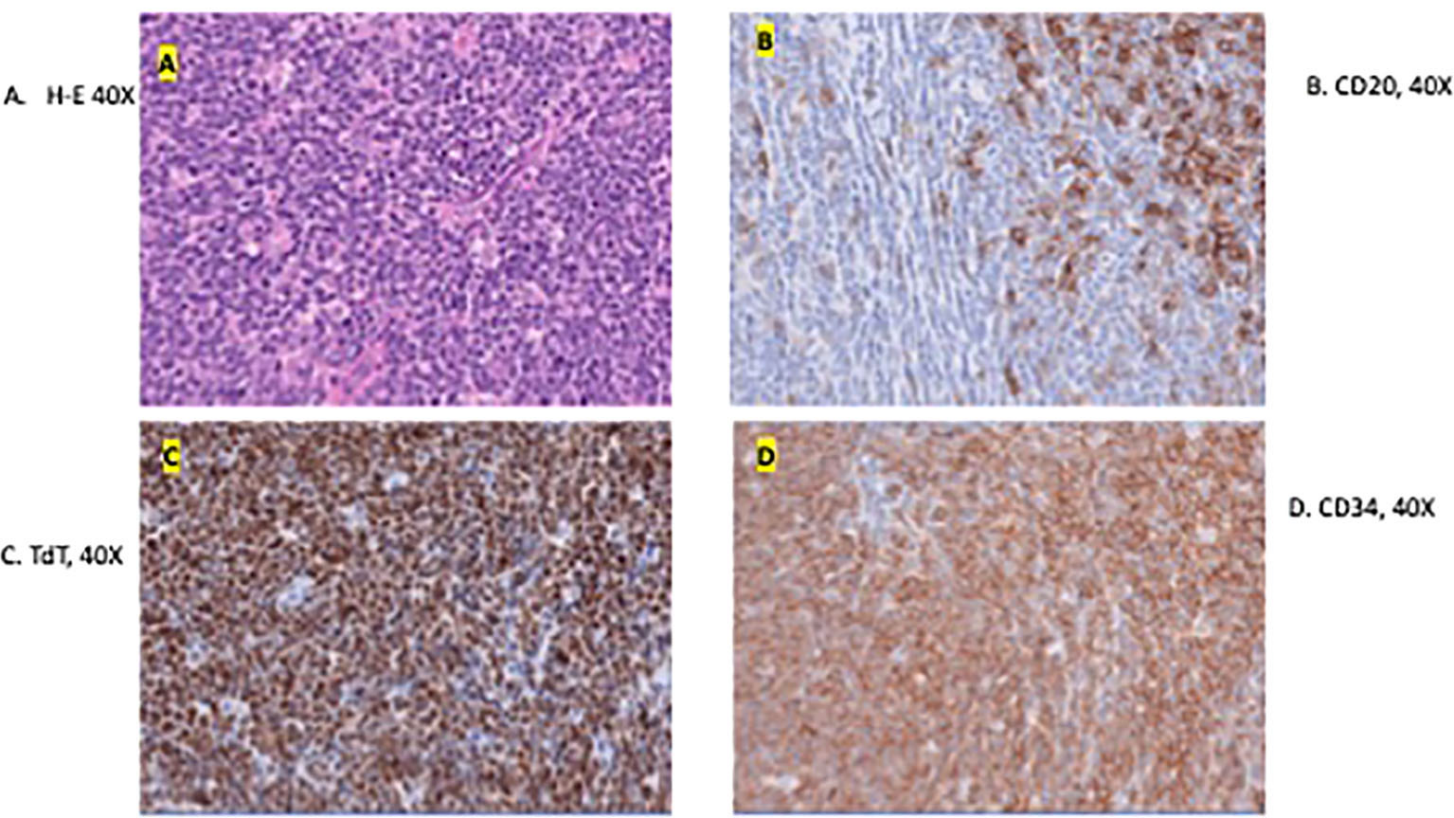
Microscopic image of the excised specimen showing dense neoplastic lymphocytic infiltration in the surround adnexal and perivascular regions and small to medium in size with irregular nuclear contour **(A)**. **(B–D)** shows the presence of tumor markers CD20, TdT, and CD34 respectively. These are indicative of a B-LBL diagnosis.

The excision specimen was a 5.5 x 4.3 cm, unoriented skin with the underlying lesion (**Figure 2**). Microscopically, it showed a dense neoplastic lymphocytic infiltration in the surrounding adnexal and perivascular regions, to the deep and superficial dermis. The neoplastic cells were small to medium in size with irregular nuclear contour. In some areas, the neoplastic cells had increased mitosis and relatively fine chromatin. Immunophenotypically, the neoplastic cells were positive for CD10, CD19, CD22, CD34, CD99, TdT, and Bcl-2, and negative for Bcl-1, Bcl-6, cMYC, MUM-1, CD30, or P53. CD20 was partially positive in the neoplastic cells, (**Figure 3**) and CD21 highlighted a residual follicular dendritic cell meshwork. Ki-67 was about 70-90%. Therefore, this was an immature B cell lymphoma, consistent with B-lymphoblastic lymphoma, rather than a mature B cell lymphoma which was diagnosed on a small punch biopsy due to the positivity of CD20. Immature markers, such as CD34, TdT were performed on the skin punch biopsy retrospectively and, they were positive. Unfortunately, these two markers were not evaluated initially, therefore, a mature B cell lymphoma diagnosis was rendered. A lumbar puncture (LP) was performed, and no neoplastic cells were identified. Subsequently, a staging bone marrow was evaluated, which only showed a mildly hypocellular bone marrow and no morphologic or immunophenotypic evidence of B-lymphoblastic lymphoma involvement.

**Figure 4:**
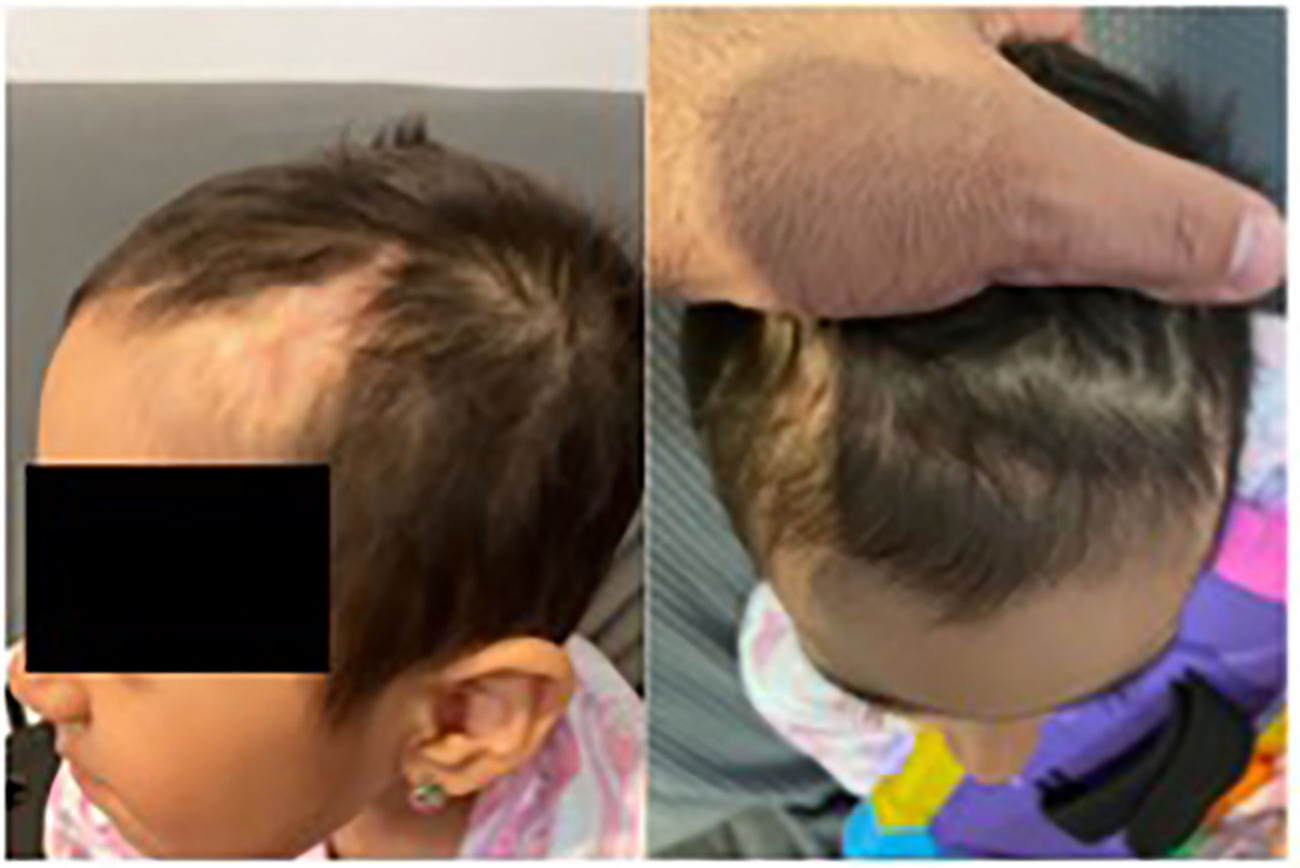
Image in 2023 of the anterior skull showing that the lesion was cured.

Unexpectedly, pathology revealed the lesion to be consistent with B-cell lymphoblastic lymphoma. The biopsy showed that the neoplastic cells were positive for CD10, CD19, CD22, CD34, CD99, TdT, and Bcl-2, and negative for Bcl-1, Bcl-6, cMYC, MUM-1, CD30, or P53. CD21 highlighted a residual follicular dendritic cell meshwork. Ki-67 was about 70-90%. A lumbar puncture (LP) and bone marrow evaluation was performed, which was negative for CNC or marrow disease. The patient underwent placement of a central venous access device (left subclavian vein subcutaneous port placement) and was initiated on treatment for Low Risk B-lymphoblastic lymphoma with a 2-year course of chemotherapy including vincristine, dexamethasone, pegasparaganese, doxorubicin and cytarabine per standard of care Children’s Oncology Group trials. She completed induction chemotherapy with minimal complications. At the end of induction, PET/CT was negative for residual disease, and she was considered in remission. Currently, she continues treatment and is clinically doing well. Cosmetically, her scalp healed well without any wound healing issues. She has a few areas of alopecia; however, the appearance of the scalp overall is excellent given her preoperative appearance (**Figure 4**). Consent was obtained from the family for the presentation of this case.

## Discussion

B-LBL is a non-Hodgkin’s lymphoma that originates in lymphoblasts and appears in about only 10% of the LBL cases (9). Unlike ALL, LBL has limited bone marrow involvement (10). The proliferation of the lymphoblasts of B-cell origin manifests primarily in lymph nodes and extranodal sites. Frequent extranodal sites are lytic bone lesions and cutaneous involvement (10–12). LBL develops more frequently in children and young adults (10). The conventional treatment for LBL uses the standard intensive, multi-drug ALL chemotherapy protocols, which includes systemic and CNS directed therapy. The implementation of pediatric-derived, intensive lymphoblastic leukemia-like protocols has produced survival rates of approximately 70% in adults and 90% in children (10, 13). Common sites of relapse for LBL are mainly the pleura, pericardium, central nervous system (CNS), liver and spleen, and to a lesser extent the skin and kidneys (it is important to note that this case is classified as a cutaneous lesion rather than a CNS site involvement). A frequency of LBL relapse in children is 25% (14). The treatment of relapsed/refractory (r/r) LBL is much more complex, and survival rates in these patients are as low as 10%. Thus, early diagnosis and definitive treatment are paramount for the survival of these patients.

This patient was initially diagnosed with Stage 1 Follicular Lymphoma based on punch biopsy of the skin. Curative therapy for these lesions in pediatrics includes gross total resection. However, pathology from our resection resulted as B-LBL, making this case unusual and mandating intensification of therapy. B-LBL lesions do not need to be surgically resected as they are extremely chemoresponsive, and treatment for LBL is similar to acute lymphoblastic leukemia. Given the risk of recurrence and relapse, systemic chemotherapy and preventative CNS therapy is required in all cases.

In general, malignant scalp masses need careful attention and treatment due to their aggressive nature (15). Multimodality imaging helps identify the type of scalp masses and differentiate between benign and malignant ones. Additionally, imaging provides information on the extent of the invasion into the scalp and progression of the disease along with aiding the care team during any operation or biopsy (16). Although complete resection of the tumor has been a primary treatment for lymphoma of the cranial vault, a meta-analysis has suggested chemotherapy and radiation also had high remission rates, regardless of the occurrence of surgical intervention. In our case, the patient’s mass continued to grow despite outside treatment from her oncologist and, therefore, underwent aggressive surgical resection (17).

In a similar B-LBL case, there was a 5x5 cm excised lesion that was treated using an anterolateral thigh (ALT) flap (8). Free flaps from the latissimus dorsi (LD) and the ALT are the most common types of reconstruction of the scalp. These have been the gold standard for large and complex lesions (18). The ALD flap particularly has been proven to be effective for scalp lesions because of its good pliability, relatively thin skin, and wide and long skin territory (19). While free tissue transfer is relatively successful, however, these approaches require separate incisions with additionally morbidity at the donor sites. Additionally, flap failure can have catastrophic consequences, potentially resulting in patient morbidity, prolonged hospital stays and, therefore, increased financial burden, and increasingly limited options for further reconstruction. Finally, success is entirely dependent on a continuous arterial inflow and venous outflow until neovascularization occurs; thus, there is significant risk to the viability of the flap if vascularization is compromised (20). There are substantial benefits to our approach if skin coverage will be feasible via rearrangement of the remaining scalp.

Our case used a rotational flap, commonly known as the O-Z flap, for reconstruction of the scalp. In general, rotation flaps are most used for lesions on the lateral face, cheeks, chin, and scalp and when other simpler types of closure fail to provide adequate functional or cosmetic results (21). It has shown to be an effective technique for wound repair after scalp tumor resection because it offers flexible design, good blood circulation, uniform tension, and good hair growth after operation (22).

Alternative treatment for scalp coverage has been tested for full-thickness scalp defects. Integra bilayer wound matrix is a technique that has a high success rate. A study showed that the patients had no wound breakdown or wound-healing issues before radiotherapy. It provides an alternative to local flaps because the scalp’s poor mobility can make it difficult to use local flaps for larger defects, even if the treatment is effective for small lesions (12).

## Conclusion

We report a unique case of a six-year-old girl with a history of a nontender scalp mass that was first noticed as a singular nodule. She was monitored for several years with insidious lesional growth, necessitating further workup. After resection of this scalp lesion, her biopsy indicated a LBL diagnosis. There are only 38 other cases in literature of patients diagnosed with B-LBL presenting with scalp lesions. Following resection, our patient did not receive a free flap reconstruction of this site, which has been the conventional method for wound repair after scalp tumor resection. Instead, we performed a rotational flap on the patient, which has substantial benefits for post-operative recovery. Our patient has been healing well and, besides a few areas of alopecia, her overall scalp cosmesis is excellent. Moving forward, in cases of scalp tumor resection, rotation flaps should be considered first when feasible before traditional free flaps, as they provide clear cosmetic and functional benefits. Finally, more work should be supported to better understand the behavior and response to systemic therapy of B-LBL scalp lesions, given the rarity of these cases and the poor survival rates with recurrence.

## Data availability statement

The original contributions presented in the study are included in the article/supplementary material. Further inquiries can be directed to the corresponding author.

## Ethics statement

Ethical approval was not required for the study involving humans in accordance with the local legislation and institutional requirements. Written informed consent to participate in this study was not required from the participants or the participants' legal guardians/next of kin in accordance with the national legislation and the institutional requirements. Written informed consent was obtained from the patients for the publication of any potentially identifiable images or data included in this article.

## Author contributions

TK, GJ, EB-W, RF were involved in the composition of this manuscript and GL, AT, YW, OT, and AK were involved in revision and review of this manuscript. TK and AK developed the concept for this report. All authors contributed to the article and approved the submitted version.
